# The suffering of chronic pain patients on a wait list: Are they amenable to narrative therapy?

**DOI:** 10.1080/24740527.2017.1316173

**Published:** 2017-07-06

**Authors:** Eloise C. J. Carr, Graham McCaffrey, Mia Maris Ortiz

**Affiliations:** University of Calgary, Faculty of Nursing, University of Calgary, Calgary, Alberta, Canada

**Keywords:** chronic pain, narrative therapy, pain management

## Abstract

**Background**: Chronic pain affects one in five Canadians. People with chronic pain frequently experience loss in their lives related to work, relationships, and their independence. They may be referred to a chronic pain program, which aims to strengthen coping through medical intervention and self-management skills. Data suggest that, even when individuals begin their pain program, many feel overwhelmed and do not continue.

**Aims**: The aim of this study was to conduct a needs assessment to explore the acceptability and feasibility of developing a psychosocial intervention, narrative therapy (NT), to address loss for chronic pain patients on the wait list of a chronic pain program.

**Methods**: Two focus groups were conducted with ten patients who had experienced being on a wait list for a provincial chronic pain management program (CPMP). Transcribed interviews were subjected to thematic and interpretive analysis.

**Results**: Two major themes emerged from the analysis: loss of identity and sharing a story of chronic pain. All patients were enthusiastic toward an NT intervention, although individual preferences differed regarding mode of delivery.

**Conclusions**: Loss is a significant part of the chronic pain experience. NT seems to be an acceptable intervention to address loss for patients on the wait list for a chronic pain program.

## RÉSUMÉ

**Contexte**: La douleur chronique affecte un Canadien sur cinq. Les personnes qui souffrent de douleur chronique vivent souvent des pertes associées à leur travail, leurs relations et leur indépendance. Elles peuvent être référées à un programme de douleur chronique visant à renforcer leurs mécanismes d’adaptation par le biais d’une intervention médicale et d’habiletés d’autogestion. Les données suggèrent que même lorsqu’un individu commence un programme de gestion de la douleur, nombreux sont ceux qui se sentent accablés et qui l’abandonnent.

**Objectifs**: L’objetif de cette étude était d’évaluer les besoins afin d’explorer l’acceptabilité et la faisabilité d’une intervention psychosociale, la thérapie narrative, abordant la thématique des pertes chez les patients souffrant de douleur chronique inscrits sur la liste d’attente d’un programme de douleur chronique.

**Méthodes:** Deux groupes de discussion réunissant 10 patients qui avaient été inscrits sur la liste d’attente d’un programme provincial de gestion de la douleur chronique ont été menés. Les entrevues transcrites ont été soumises à une analyse thématique et interprétative.

**Résultats:** L’analyse a permis de dégager deux thèmes principaux: la perte d’identité et partager une histoire commune de douleur chronique. La thérapie narrative a suscité l’enthousiasme de tous les participants, bien que leurs préférences différaient en ce qui concerne les modalités de mise en œuvre de l’intervention.

**Conclusions:** Les pertes sont un aspect important de l’expérience de la douleur chronique. Pour les patients inscrits sur la liste d’attente d’un programme de douleur chronique, la thérapie narrative semble être une intervention acceptable pour aborder cette question.

## Introduction

One in five Canadian adults suffers from chronic pain, with a prevalence rate of up to 30% across Canada^1^ and approximately 35% of adults in Alberta.^2,3^ *Pain* has been defined as “an unpleasant sensory and emotional experience associated with actual or potential tissue damage, or described in terms of such damage” and is usually described as chronic after 3 months.^4,5^ Chronic pain often results in significant suffering, disability, and reduced quality of life,^3^ because it affects sleep, mood, anxiety, cognition, and emotional functioning.^6^ In addition to the significant personal burden,^7^ the societal economic costs are estimated to exceed $6 billion.^8^ To focus treatment on the pain component and ignore the patient’s depression, anxiety, loss of self-esteem, and inability to return to work can cause more harm than not treating the patient at all.^9,10^

Initial care for chronic pain occurs in primary care settings; those with more severe or refractory pain are usually referred to a chronic pain program (CPP). Systematic analysis shows that chronic pain multidisciplinary pain programs in tertiary care are effective.^9^ However, the provision of tertiary care CPPs in Canada is inadequate. Canada’s priority areas for wait time targets do not include chronic pain, and the majority of pain sufferers still experience unacceptable wait times to receive services.^11,12^ This is consistent with data from the Calgary CPP, which demonstrates that patients wait 9–12 months to receive services.^13,14^

Canadians waiting more than 6 months for access to appropriate chronic pain treatment are more likely to see deterioration of their quality of life and psychological well-being, including higher depression scores.^2,15^ Studies show that chronic pain can double the risk of death by suicide^16^ and that uncontrolled pain compromises immune function, promotes tumor growth, and can slow healing, with associated increases in morbidity and mortality following surgery.^17^ Patients on lengthy CPP wait lists often fail to attend or engage once admitted to the program.^18,19^ For example, the Calgary CPP reports that up to 50% of patients withdraw after initial orientation.^13^ This suggests a lack of preparedness for the chronic pain program, which emphasizes self-efficacy and readiness for change.^20^

Chronic pain is associated with a variety of life issues causing despair, and the pattern of despair often includes personal stress, psychological disturbances such as depression, maladaptive and dysfunctional behaviors, and social isolation.^16^ Chronic pain sufferers frequently experience a significant sense of loss related to occupational abilities, social networks, relationships, future plans, as well as poor physical function and reduced quality of life.^21^ Studies suggest that loss caused by chronic pain may lead to grief experiences and unbearable suffering.^22,23^ Treatment interventions to ameliorate grief have been shown to be effective in a range of different patient populations, including mental health^24^ and cancer.^25^ Expressing grief and becoming aware of these losses is an important part of self-understanding.^26^ For patients with chronic pain, it may be that acceptance of chronic pain opens the doors to working effectively on acknowledging that life has changed and that there are losses to accept and integrate into one’s life story. Interventions such as writing therapy, group and individual support through narrating story, and ventilating feelings are reported as helpful to ease the loss experience.^27^

Narrative therapy (NT) is a therapeutic tradition focusing on “reauthoring” stories of suffering into stories of resiliency and efficacy^28,29^ and has been practiced for over 30 years with a range of populations, including patients with major depression,^30^ eating disorders,^31^ family relationship problems,^32^ and chronic pain.^33^ NT starts from the idea that we all have stories about our lives that we have accrued over time by linking together events in our lives into a meaningful sense of who we are. When problems that affect our lives become part of the story, NT can help to work out ways of making sense of what is going on that can be more beneficial in dealing with the problem.^34^ In practice, “NT treats people as experts on their own lives, is a non-blaming approach, and views problems as separate from people”(p. 2).^35^

The aim of this pilot study was to undertake a needs assessment to inform the development and testing of a psychosocial intervention, NT, to ameliorate the effects of loss and grief as experienced by people with chronic pain on a wait list for a chronic pain program. The research objectives were to

1. understand the experiences of people with chronic pain around the concept of loss.
2. explore the acceptability of an NT intervention, to address loss, for patients in terms of content and feasibility.
3. explore which outcomes patients identify as being important to them when evaluating the potential impact of an NT intervention.
4. explore the acceptability of the mode of delivery for the NT intervention: face-to-face; telephone, online, or a blend of modalities.

## Methods

### Research design

Qualitative methods were most appropriate for pursuing the aims of this study and to gain insight into the experiences of people with chronic pain. This qualitative study formed the first phase (pilot) of three distinct studies in a planned program of work around loss and chronic pain.

### Data collection methods

Focus groups were used to collect the qualitative data because they encourage exploration of a particular topic in an informal setting.^36^ Focus groups are compatible with a range of research methods by generating rich data around a given topic that may be analyzed in complementary ways.^37^ For this pilot study, two focus group sessions were conducted.

### Sample

This study sought a purposive sample of eight to ten individuals per focus group who had been referred to the CPP in Calgary. It has been found that a sample of ten to 12 similar people enhances the quality of the data.^38^

#### Inclusion criteria

Patients had to be 18 years of age or older, to have had chronic pain present for 6 months or longer, measuring 4 or higher on a visual analogue scale (0–10 mm) at the time of invitation to the study. Patients had to provide informed consent and be able to converse in English.

#### Exclusion criteria

The main exclusion was presence of pain for less than 6 months or the presence of other serious health concerns that could impact on the notion of loss, such as severe immobility, cardiopulmonary disease, and current neoplasm.

### Data collection

Following ethical approval from the University Conjoint Health Research Ethics Board (#REB13-0940), data were collected between July and September 2014. Eligible patients were invited to participate in the study when the letter from their referring family physician to the Calgary CPP had been acknowledged and the patient had been formally invited to attend the orientation to the program. An advertisement was also posted in the CPP Centre featuring the study details. The focus group was conducted at the Calgary Pain Centre, using semistructured interview questions, and explored the following topics: (1) understanding the experiences of people with chronic pain around the concept of loss; (2) exploring the acceptability of an NT intervention; (3) exploring which outcomes patients identify as being important to them when we evaluate the potential impact of an NT intervention; (4) exploring the acceptability of the mode of delivery for the NT intervention: face-to-face, telephone, online, or blend of modalities. A sample interview schedule is provided in the Appendix. We ensured that patients had access to counseling services, should they become distressed. However, previous experiences and research suggest that many chronic pain patients gain support from sharing their stories.^39^

### Data analysis

Qualitative data from the digitally recorded focus groups were transcribed. An inductive content analysis was used to allow a rich understanding of the themes embedded in the responses to the open-ended questions.^40^ The purpose was to reduce the content to provide a condensed but broad description of the data. The text was read thoroughly; words and short statements were identified that captured meaning. These codes were then grouped into similar topics and finally collapsed into subthemes and themes. E.C. and M.O. coded the data and identified subthemes and themes. All authors discussed and agreed on the final themes.

### Ethical considerations

Participants were asked to recount their experiences of pain and explore the concept of loss that might have evoked feelings of sadness and distress, through the retelling of their own personal stories about pain. The facilitator was an experienced nurse and was sensitive to these issues. Participants were told that they could withdraw at any time and were provided the name and number of a local psychological counseling service.

## Results

Ten patients participated in two focus groups (*n* = 6, *n* = 4), including seven women and three men. The youngest participant was 40 years and the oldest was 65 years, with an average age of 49 years (SD = 7.8). They had all experienced chronic pain for between 3.5 and 36 years, with an average length of 12.75 years. The reason for their chronic pain varied, with four participants having back pain but others citing complex regional pain syndrome, fibromyalgia, migraines (two), myofascial pain, and neuropathic pain. When asked to provide a current pain score using a 100-mm visual analogue scale with anchors of *no pain* and *worst pain ever*, the average pain intensity score was 75. It was evident that participants experienced significant and enduring chronic pain.

Participants readily shared their experiences of chronic pain, which centered on loss and grief, and expressed the importance of sharing pain experiences and the provision of validation. This alludes to both the natural fit of NT as an intervention and its prospective benefits. Two major themes emerged from the analysis: loss of identity and sharing a story. Modalities of therapy were also discussed; participants disclosed their opinions on face-to-face, telephone, online, or a mixture of service delivery.

### Loss of identity

One of the major themes that emerged from the focus group data was how chronic pain threatened the patients’ sense of identity. The theme of identity has intrapersonal, interpersonal, and temporal dimensions that accord with the insight that a stable and flourishing sense of personal identity is a narrative achievement.^27,41^ Participants talked about losses including aspects of functioning, career, plans for the future, and family roles. The sheer fact of loss was often accentuated by how others responded to them and perceived them, leaving them feeling unheard and unvalued. One participant described that the effect of her pain on functioning that had the biggest impact on her life:

Function is more important to me than pain is. Because the pain is there, it’s always going to be there, but how much I can do … is what’s important. […] And not just within the home. Your function outside, your social activities, your interactions with other people, even picking up the phone to call a friend. (Participant P)

Another participant talked about her difficulties doing housework through the lens of her son’s reaction:

I’ve tried to talk to my son about it, um. Because, he, um, he used to get really angry at me or couldn’t understand why I was, um, resting more than usual. And I wasn’t doing the housework, or I wasn’t getting the dishes done, or I couldn’t stand in the kitchen long enough to make meals. (Participant S)

In this example, it was not just the loss of functioning that was experienced but the loss of role within the family. Another participant expressed her own sense of loss of a previous identity through listing activities she used to enjoy:

But everything was so foreign to what I was then before. I was fit, I was able to do things, I was able to ride my bike, run, walk, or … a number of things. My body worked fine, ah, … then, your body quits working. (Participant H)

Identity is a consistent sense of self across time; it depends not only on looking back to who we used to be but also projecting a continuation of stable identity into the future. Other participants drew attention to how the anticipation of continuing identity was disrupted by their chronic pain:

… our accident actually happened right, three months after we got engaged [laughs]. So, I mean, ah, you know, when you, first, when you … think that, you have, people get engaged and they have thoughts of what their life is going to be like, right? … You know, you don’t realize how, what an impact it can have on you, um, until you’re actually living through it. And going through that realization that “yeah, this is so not what I thought it, life was going to be like four years ago.” (Participant LA)

Participants expressed that another painful part of the loss of identity was finding that others could not adjust their expectations or even imposed their own ideas about who participants now had become:

My, my experiences are very similar to everybody here [in the focus group]. … I have two brothers who do not want to hear it, and if you do start to talk about it they’ll actually physically leave the room. My dad, […] “Why don’t they fix you; you’re not doing what you’re supposed to do; it’s all about you, you, you, you; you’re not getting better, what are you doing wrong?” (Participant P)

Another participant that she was labeled by others as a chronic complainer:

I think I was viewed as … not someone who had chronic pain, but in their eyes, I was a chronic complainer now. Coming from a positive, outgoing, I don’t … happy-go-lucky type person and now. … “You’re a chronic complainer, that’s why you always complain about your pain. I can’t handle you.” (Participant H)

In these painful experiences, participants described the ways in which chronic pain affected their sense of themselves in relation to their past and future expectations for themselves, as well as in relation to others. Pain intruded into the participants’ lives, interrupting their personal stories of who they were and what mattered to them. The perspective of identity-as-narrative, however, also offers possibility for restitution, for regaining some control of the story. In other words, their loss of identity may be amenable to change through the retelling of their pain stories in the future.

### Sharing a story

The second major theme captured the significance of sharing a story and included several interrelated aspects. Participants talked about the importance of someone listening to them with empathy and believing them. Most mentioned the importance of a support group that provided an opportunity to share a story about pain and also experience the realization that they were not alone. Finally, the opposite, where the detrimental effects of not being believed, particularly by health professionals, was experienced by some.

Participants identified empathy and being believed as aspects of being listened to that were very important to them. Sharing their pain stories, where others actively listened, believed them, and demonstrated empathy, was a profoundly supportive experience:

I had one friend that would sit with me and not say a word. And I could dump all over him and you feel great, after, you feel great. But you feel so much better, that someone listened. He couldn’t help me, as far as, like, medical, or intervention or any kind of therapies, but he was such a friend. And, and that meant, to this day, it meant so much to me. That someone was there to just listen and, and didn’t offer opinions, didn’t, “Oh, you haven’t tried this,” “You need to try this.” No, he was just a sounding board. (Participant H)

Several participants talked of sharing these stories with friends who had a medical background or had experienced chronic pain themselves:

I’ve been more fortunate, I’m lucky to say. So I’m a family doctor, and, and it’s, I’m not going to say it’s easier but a lot of my close friends are more medical. … I can see that they can truly understand, um, and just, it’s nice to just talk about it and not, you know, just to kind of let it off of your chest and, and to have somebody truly say, “Yeah, that really sucks,” or, you know [laughs]. (Participant LA)

I found it was easier to tell people who had pain themselves. […] Like my friend who has steel rods in her back. It was easier to tell her than … someone who doesn’t get pain. There was more empathy and not being dismissed. (Participant L)

However, the impact of an encounter when sharing their stories of pain and not being believed could be very negative from colleagues, friends, or health providers:

… and then I had people say that, “you look fine to me.” Um, or, “You don’t want to work,” you know, even after I’ve had a very successful career, so it was very insulting to me. (Participant CL)

It would’ve been helpful to me [to have had a health professional listen to my story], because unfortunately my family doctor was not like you [to Participant LA] and she kept telling me it was psychosomatic, and it was all in my mind, and … because of the stuff I’ve been going through. … (Participant CY)

Experiencing chronic pain and being able to share that story with others appears to be a central tenet for people living with pain. The need to be believed and having the listener empathize appears to be a supportive experience.

### Patient outcomes and mode of delivery

We asked participants which outcomes would be important for them if we were to evaluate the impact of NT on their pain experience. Patients suggested psychosocial outcomes to include mood, self-esteem, and hope. A suggestion was made to take into account the impact of the season on these outcomes; for example, the effects of cold during winter months.

There was significant variation in preferences for mode of delivery: face-to-face (two), telephone (two), online, or a blend of modalities (three), with three indicating no specific preference. Pros and cons were mentioned for all modalities; for example, telephone and Internet allow more flexibility.

## Discussion

The aim of this pilot study was to undertake a needs assessment to inform the development and testing of a psychosocial intervention, NT, to ameliorate the effects of loss and grief as experienced by people with chronic pain. The focus group was around the notion of loss and chronic pain, and the opening questions provided a catalyst to share many stories of their own experiences of loss related to chronic pain. It was evident that none of the participants struggled to talk about loss or needed to be coaxed into telling a story of loss, because the richness and diversity of these losses were immense; telling a story was a very natural way of gaining support and understanding.

The importance of sharing the story was a major theme. The study showed the importance of allowing patients to tell their stories from their own points of view, rather than as a medical history where the professional often dictates the form of expression, the order of the telling, and what counts as relevant.^27^ Equally important, the participants’ accounts conveyed the significance of stories as social events, requiring both a narrator and an attentive listener or listeners. When participants had not felt heard or that their stories were labeled and dismissed as complaining, it accentuated the pain of lost identity and isolation. In contrast, they spoke positively of friends or relatives who were able to stay with the story of pain and, as a result, they felt more connected and confident in undergoing the adjustments in identity forced upon them by their pain. It is important, therefore, to keep in mind that storytelling is a social process in which health care professionals participate, with real effects—for better or worse—on their patients. Health care professionals may feel impotent to help patients with chronic pain, but perhaps listening to their stories can be seen as a therapeutic intervention. Nonpharmacological comfort measures have been identified as particularly important for nurses, who often feel disempowered to manage pain.^42^

The focus groups explored the question of whether patients on a wait list for a chronic pain program thought that NT would be a suitable intervention. The ways in which participants responded positively to the specific question, as well as demonstrating the value for them of telling their stories in a supportive environment even in the focus groups, suggests wider implications for health care providers. For example, in routine interactions with patients suffering with chronic pain, professionals such as physicians, nurses, or physical therapists can make/create positive interventions simply by listening for themes of loss and identity adjustment and showing recognition of each patient’s situation. In a qualitative systematic review of experiences of patients with chronic pain, the authors found that the experience of health care was often adversarial and concluded that “affirming a person’s experience and allowing an empathetic interpretation of their story is not an adjunct, but integral to care” (p. 835).^43^ There continues to be emphasis on initiatives to improve patient–physician communication, because better communication results in improved patient outcomes.^44^

Our second theme around loss of identity emphasized the significant losses experienced by these people across multiple aspects of their lives. Loss of identity across the social roles of occupation, leisure, friends, and family has been well reported in the chronic pain literature.^45–47^ Our study captured the significant loss for those with chronic pain, as it related to the past, the future, and the present. It has been suggested that chronic pain can threaten not only current self-identity but also the future of who one might become.^48^ We see NT offering the opportunity to reauthor these stories and provide participants with some control of the current and future narrative, thus providing support for them while on the wait list for a chronic pain program.

It is important to also note that an individual’s narrative can have recursive aspects, especially when relating a significant life change, such as chronic pain, in the context of interpersonal relationships. Interpersonal networks, principally family systems, and their processes are best comprehended in the view of circular versus linear causality.^49^ In this regard, NT would aid participants in identifying and better understanding the complex changes to their interpersonal networks in relation to their chronic pain experience and facilitate improved communication, rather than trying to apportion a direct cause or blame.

Participants supported the suggested outcomes when evaluating NT but emphasized the psychosocial impact in particular, reflecting the multidimensional nature of pain. They also discussed the pros and cons of different delivery modalities, with little consensus for a preference. There is evidence to suggest that people experiencing pain may find it easier to talk about their pain and disclose their feelings via the telephone,^50,51^ and there is strong support for the adoption of online therapeutic psychological interventions.^52^ It is unlikely that face-to-face would be cost effective, but offering a choice of telephone or online delivery would be a viable alternative to meet individual preferences. Telephone delivery of a psychological intervention has been shown to offer improved adherence and less attrition compared to face-to-face delivery,^53^ perhaps an important consideration because many patients on a chronic pain program wait list do not engage with the program once it is offered.

## Limitations

The number of participants in the focus group was less than anticipated, although we observed rich and meaningful discussions that might be seen as a trade-off for the (limited) numbers.^54^ The participants all experienced a chronic pain program and had likely been exposed to health professionals who believed their experiences around pain. This may have given rise to a more positive perspective of their interactions with health professionals than those reported in the literature.

## Conclusion

This study identified how patients with chronic pain told their pain stories and the importance of loss on multiple aspects of their lives, past, present, and future. Though aspects of loss of identity have been explored, there are indications that this warrants further research to gain greater understanding. NT was welcomed as an intervention that was perceived as potentially helpful with the adjustment to loss while on a wait list for a chronic pain program. The next steps will be to develop an NT intervention and evaluate its effectiveness on patient engagement with a chronic pain program and outcome measures identified as important by these patients, such as mood, self-esteem, and hope. In addition, this study emphasizes the central role of communication, giving health care professionals opportunities to support patients by listening to their stories.

## Appendix—Focus group interview schedule

1. Introduce idea of narrative therapy with description and invite any questions or clarification from participants about it.
Narrative therapy starts from the idea that we all have stories about our lives that we have built up over time by linking together events in our lives into a meaningful sense of who we are. It is based on the idea that telling our stories to each other is a normal and natural part of social life and of supporting each other. When problems that affect our lives become part of the story, narrative therapy can help to work out ways of making sense of what is going on that can be more beneficial in dealing with the problem. Narrative therapy treats people as experts on their own lives, is a non-blaming approach, and views problems as separate from people (p. 11).^1^

We hope to use results of this study to help gauge the feasibility of a larger study that will use narrative therapy.

2. Questions about previous experience of “narrative”:

(a) Can you think of a time when you have told someone else the story of your pain, how it entered your life, and what effect it has had on you? Who have you told it to?
(b) What has it been like for you to put together the experience in this way?

3. Questions about possible narrative intervention:

(a) At this point, maybe reemphasize that narrative therapy would be different in that the therapist would be looking for ways of helping you to “reframe” the story.

(b) As part of coping with having to wait to be seen at the pain clinic, do you think you would find it helpful to have an opportunity to share your experience of pain in this kind of way with a health care professional?

(c) There are a number of possible ways of communicating your experience, and I would like to ask you about the pros and cons of each of them.

- What do you think the advantages might be for you of talking to someone face-to-face?
- What do you think the disadvantages might be for you of talking to someone face-to-face?
- (Same paired questions for telephone or online.)
- Prompts about practicalities; for example, transport if face-to-face, computer access.

(d) Based on what I have been able to describe to you today, can you say which way of communicating you think would be the most helpful for you?

Adapted from Morgan.^35^
